# A pilot study combining individual-based smoking cessation counseling, pharmacotherapy, and dental hygiene intervention

**DOI:** 10.1186/1471-2458-10-348

**Published:** 2010-06-17

**Authors:** Semira Gonseth, Marcelo Abarca, Carlos Madrid, Jacques Cornuz

**Affiliations:** 1 Department of Ambulatory Care and Community Medicine, Lausanne University Hospital, 1011 Lausanne, Vaud, Switzerland

## Abstract

**Background:**

Dentists are in a unique position to advise smokers to quit by providing effective counseling on the various aspects of tobacco-induced diseases. The present study assessed the feasibility and acceptability of integrating dentists in a medical smoking cessation intervention.

**Methods:**

Smokers willing to quit underwent an 8-week smoking cessation intervention combining individual-based counseling and nicotine replacement therapy and/or bupropion, provided by a general internist. In addition, a dentist performed a dental exam, followed by an oral hygiene treatment and gave information about chronic effects of smoking on oral health. Outcomes were acceptability, global satisfaction of the dentist's intervention, and smoking abstinence at 6-month.

**Results:**

39 adult smokers were included, and 27 (69%) completed the study. Global acceptability of the dental intervention was very high (94% yes, 6% mostly yes). Annoyances at the dental exam were described as acceptable by participants (61% yes, 23% mostly yes, 6%, mostly no, 10% no). Participants provided very positive qualitative comments about the dentist counseling, the oral exam, and the resulting motivational effect, emphasizing the feeling of oral cleanliness and health that encouraged smoking abstinence. At the end of the intervention (week 8), 17 (44%) participants reported smoking abstinence. After 6 months, 6 (15%, 95% CI 3.5 to 27.2) reported a confirmed continuous smoking abstinence.

**Discussion:**

We explored a new multi-disciplinary approach to smoking cessation, which included medical and dental interventions. Despite the small sample size and non-controlled study design, the observed rate was similar to that found in standard medical care. In terms of acceptability and feasibility, our results support further investigations in this field.

**Trial Registration number:**

ISRCTN67470159

## Background

Tobacco use is one of leading causes of preventable mortality in industrialized countries [1]. Only 0.5 to 3% of smokers who initiate a spontaneous attempt to quit smoking stay abstinent after 12 months [2]. An attempt to quit cigarettes with help from a specialized team and nicotine replacement therapy gives, on average, a 17% rate of tobacco abstinence after one year [3]. New strategies to improve the smoking cessation rate during attempts to quit are strongly needed.

Dentists have regular contact with smokers and a great potential for helping their patients to quit smoking; yet, this potential is often underused [4-14]. The point at which a dentist finds periodontal lesions related to smoking could be a *teachable moment *to change this behavior [15,16]. Before conducting a study to assess the potential benefit of a dentist's intervention during a medical smoking cessation program, we performed a pilot study to assess its feasibility and acceptability as part of the usual care for smokers attending a smoking cessation clinic. As a secondary outcome, we measured the 6-month continuous abstinence rate.

## Methods

### Study design and participants

Study participants were recruited through public advertisements in the hospital area and in local newspapers. Interested participants were invited to call the study center. A description of the trial was provided and a pre-screening interview was made on the phone. Inclusion criteria were the following: age between 18 and 70; currently smoking for ≥ 3 years at least 10 cig./day; and a score of minimum 6/10 on the psychometric Likert's Scale in response to the question "What is today your motivation to quit smoking from one to ten?". Exclusion criteria were the following: current pharmacological use to quit smoking; presence of an unstable or life-threatening medical condition; current unstable psychiatric illness; at risk alcohol consumption; illegal drug consumption, such as cannabis; because dental hygiene treatment causes a bacteriemia, people at risk to develop an endocarditis, i.e. meeting American Heart Association's criteria for antibiotic-prophylaxis before dental intervention [17]; long-term bisphosphonate treatment; and recent oral hygiene intervention (< 6 months).

At the first visit, oral and written explanations of the trial were provided about participant's implication, risks, and benefits. Participants gave their written informed consent. Afterwards, we took detailed medical, oro-dental, and smoking histories. Anthropometric measures were obtained, i.e. arterial blood pressure, self-reported body weight and height, and carbon monoxide expiration rate.

### Study setting

The study was performed in the Department of ambulatory care and community medicine, which includes a primary care clinic and a dental clinic at the same building. This clinic is connected to the main hospital of the region, and is easily accessible by public transport.

### Smoking cessation intervention

All participants received an 8-week smoking cessation intervention including individual-based intervention combining replacement therapy and/or bupropion and 4 sessions of counseling. Counseling was based on national and international current guidelines, targeting increasing the motivation to quit smoking [18], the identification of barriers, and the prevention of relapse [19,20]. A counseling session lasted thirty minutes in average. Participants received a combination of nicotine replacement therapy (transdermal patch 16-hour/day or 24-hour/day, 1-mg or 2-mg lozenge, 2-mg or 4-mg gum, 10-mg inhaler) and/or bupropion, according to the participant's past experiences and preferences. Four visits (at week # 1, 2, 4, and 8) were scheduled and participants were asked to plan a quit date from the inclusion day until the 4^th ^visit at week 8. They were considered as smokers if they failed to quit or if they relapsed to smoking afterwards. Participants lost during follow-up were called and received a letter explaining the scientific implications and the need for follow-up, and were invited to contact us.

### Dentist's Intervention

The dental intervention was provided by a dentist trained in periodontology (MA) and included two visits. At the first visit, the dentist performed an oro-dental exam to rule out oro-dental lesions, e.g. periodontitis, gingivitis, and other oral or dental lesions. At the end of this visit, the dentist orally explained the results of the oro-dental exam, i.e. detailed explanations of the lesion(s) related to smoking, and recommended treatment if necessary. He also provided standardized information about chronic effects of smoking on oral hygiene (e.g. bad breath, esthetic sequelae), chronic effects of smoking on oral health (e.g. increased risk of oral cancers or periodontitis), and a brief explanation about periodontitis (a chronic infection of periodontal tissues, beginning with gingivitis and gingival bleeding, that is often hidden by smoking). The dentist also provided oral and illustrated explanations of dental plaque and made a practical and individualized demonstration of oral hygiene techniques, e.g. correct teeth and tongue washing, correct dental floss/sticks use. The first visit lasted about one hour. At the second visit, one week later, the dentist performed an simple oral hygiene treatment - which was not a treatment of periodontitis - using the full mouth periodontal debridement technique with an ultrasound device (EMS^®^-Air Flow^® ^S2) [21]. In terms of treatment and potential physical annoyances, results of full mouth disinfection and classic approach are similar for the patient. During this visit, a second verbal intervention reinforcing the importance and the correlation of potential periodontal and oral lesions and smoking was performed by the dentist.

### Data collection

The acceptability and feasibility of the dentist's intervention was assessed by a hetero-administrated evaluation questionnaire comprising four questions on global satisfaction, acceptability of potential physical annoyances due to the dentist's intervention, and the duration of the intervention, as well as two open-questions on advantages and disadvantages of the dentist's intervention. The questionnaire was administrated by the study staff of the smoking cessation clinic. Data were anonymous.

A follow-up visit was scheduled at 6-months for participants that were abstinent at the 4^th ^visit. The recruitment was conducted from November 2007 to May 2008. The follow-up ended in Autumn 2008. Continuous smoking abstinence was defined as self-reporting of continuous smoking abstinence from the 8-week visit to the 6-month visit, and biochemical validation by an expired carbon monoxide rate less than 10 ppm [22]. A maximum of 5 cigarettes smoked during the abstinence period was tolerated [22]. The Lausanne University's Medical School Ethics Committee approved the research protocol. The participation to the whole study was free of charge for subjects.

## Results

**Figure 1:**
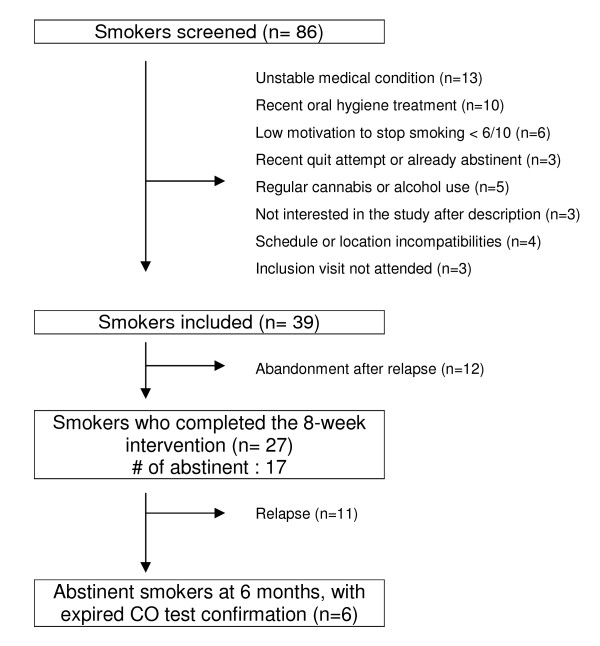
**Flow chart**.

As reported in Figure 1, a total of 86 interested people called the study center. Among them, 39 adult smokers met the inclusion criteria and were included in the study. Thirty-four attended the first three visits and 27 the fourth one (week 8) at the end of the intervention. The mean age of the subjects was 36 years (range 22-53 years), 59% of the subjects were women, the mean (SD) [kg/m^2^] body mass index was 22.4 (3.06), the mean (SD) number of daily smoked cigarettes was 18.7 (8.0), and 18% of the subjects had a high level of education. At least one previous quit attempt was made by 97% of the participants. Nicotine replacement therapy was used by 97% of the participants. At the 8-week visit, the proportion (SD) of participants using transdermal patches was 50% (9.9), lozenges or gums 57.7% (9.8), inhalers 19.2% (7.8), and bupropion 3.8% (3.8).

**Figure 2:**
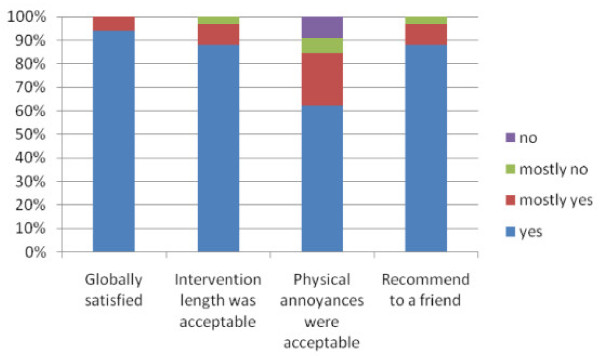
**Qualitative assessment of the dentist's intervention**.

As described in Figure 2, participants were globally satisfied with the dental intervention (94% yes, 6% mostly yes). The duration of the dental intervention was considered acceptable by most of the participants (87% yes, 10% mostly yes, 3% mostly no). The potential annoyances due to the dental intervention were considered acceptable (61% yes, 23% mostly yes, 6%, mostly no, 10% no). The majority of the participants would recommend this intervention to a friend (87% yes, 9% mostly yes, 3% mostly no).

Participants gave several positive qualitative comments about the motivational effect of the explanations on their dental and oral status related to smoking habits; for example, one participant mentioned "The dentist's explanations about the effect of tobacco use on oral health were motivating." They also reported positive comments about the feeling of oral cleanliness and health that encourages smoking abstinence; for example, one participant said "Once my teeth were clean, I did not want to spoil them." However, a few negative comments highlighted too much information was provided on tobacco related oral diseases, such as "What the dentist said about the effects of tobacco on the mouth seems to me, exaggerated."

At the end of the intervention (week 8), 17 (44%) participants were abstinent from smoking. At the 6-month follow-up visit, 6 (15%, 95% CI 3.5 to 27.2) reported a confirmed continuous smoking abstinence. The oral exam revealed the vast majority (59%) of the participants had an unhealthy oro-dental status. Eleven participants (28.2%) presented gingival inflammation, e.g. bleeding, and 12 (30.8%) had periodontitis. Moreover, 2 (5.1%) participants presented with a severe form of post adolescent/adult periodontitis. Three participants presented with a pre-cancerous lesion, such as hyperkeratosis of the tongue (n = 1) or gingival leucoplakia (n = 2).

## Discussion

The feasibility of the dentist's intervention during a smoking cessation attempt made in a smoking cessation clinic was considered appropriate. The method of participants' recruitment was suitable, since it required two advertisements in the local press and a few advertisements in the hospital area to obtain a sufficient number of participants. Our subjects were representative of the smokers from the general population in terms of age, number of smoked cigarettes per day, proportion of men and women, and scholar education level [23]. The participation rate is in concordance with those found in other smoking cessation studies (60 to 70%) [24,25]. A particularly high retention rate was obtained during the primary phase of the study. The duration of the dentist's intervention correspond to the usual time dedicated to a patient in the dental setting of our out-patient clinic. It is possible that it does not correspond to private dental settings.

Acceptability of the dentist's intervention was also considered high. Even though the participants of our study were smokers that first sought medical help for smoking cessation, we succeeded in obtaining high global satisfaction and acceptability towards the dentist's intervention. In addition, participants provided positive qualitative comments about the dentist counseling, the oral exam, and the resulting motivational effect.

Using caution due to the design of this study, i.e. a non-controlled pilot study with a small sample size, we measured a 6-month continuous smoking abstinence rate. The results were similar to the observed rates of smoking quit attempts managed in smoking cessation clinics [3]. We observed a high initial cessation rate, since almost half of participants had quit smoking 6 weeks after the second dentist intervention. Indeed, although the recruited participants had an initial motivation to quit smoking of min. 6/10 on the Likert Scale, abstaining from smoking is hard to achieve, even when smokers say that they are ready to stop, and relapse rate is high during the early post-cessation period [26]. Oral effects of smoking, i.e. esthetic sequelae, bad breath, gingival deterioration, are more visible than other smoking related health consequences, such as athero-sclerosis or pulmonary lesions. The dentist intervention helped the smokers to identify their own oral lesions due to smoking, and the oral exam revealed the majority of participants presented with oro-dental effects from tobacco. In fact, the participants described such a motivational effect in their comments. However, the design of our intervention did not allow any further contact between the participants and the dentist until the end of the study. This motivational aid was possibly missing during the consolidation stage. Additional contacts with the dentist could reinforce the motivation to stay abstinent from cigarettes and could minimize relapse during follow-up.

Almost two-thirds of the participants had an unhealthy oro-dental status, which was not surprising in smokers, despite the young mean age of our study's population [27]. The proportion of periodontitis found during the oro-dental exam was important and the proportion of post adolescent/adult periodontitis - in an aggressive form of disease of gums and alveolar bone leading to a loss of teeth- was two-fold higher than the proportion occurring in the general population [28]. It is not possible to treat periodontitis in one session, because this needs long-term treatment. Participants in whom we discovered this pathology did benefit of our intervention though, and were aware about the need of further treatment.

To improve the real-world applicability of this pilot study, some potential drawbacks should be taken into consideration. For instance, as in real practice hygienists might perform counseling and hygiene treatment more frequently than dentists, they should also be involved in this intervention. Our pilot study could also improve from the questionnaire about the acceptability of the dentist's intervention being self-administered or administered by a team independent from the study staff. This questionnaire was indeed administered by the smoking cessation team, and we cannot totally exclude a response bias.

## Conclusions

Our study confirmed the feasibility and acceptability of a new multi-disciplinary approach combining the usual smoking cessation counseling with a dentist's intervention. Moreover, given the current prevalence of periodontitis in our study population, the results of screening for periodontitis among smokers seeking help to quit smoking should be assessed in further studies. We are planning to conduct a larger study with a modified study design, including regular contacts between the participants and the dentist during the consolidation stage, to reinforce the motivational impact of the dentist intervention. Due to the length of the dentist's intervention, which might be too demanding especially in private dental settings, and its cost, we are planning to include the dental hygienists to perform the oral hygiene treatment.

## Competing interests

The authors declare that they have no competing interests.

## Authors' contributions

SG participated in the conception, the design and the coordination of the study, carried out the smoking cessation intervention, performed statistical analyses and drafted the manuscript. MA participated in the design of the study and performed the dentist's intervention. CM participated in the conception and the coordination of the study, and performed the supervision of the dentist's intervention. JC participated in the conception, the design and the coordination of the study, and carried out the supervision of the smoking cessation intervention. All authors read and approved the final manuscript.

## Pre-publication history

The pre-publication history for this paper can be accessed here:

http://www.biomedcentral.com/1471-2458/10/348/prepub
